# Davis and Hahnemann

**Published:** 1900-03

**Authors:** Henry R. Hopkins

**Affiliations:** 433 Franklin Street. Buffalo


### Correspondence.

### DAVIS AND HAHNEMANN.

*To the Editor:*

*Sir*—We hear from a recent correspondent of one of our most influential metropolitan dailies, that a joint resolution of medical interest has recently passed both houses of Congress and received the signature of the president. This resolution grants permission for the erection of a monument in honor of Samuel Hahnemann at some suitable place in the City of Washington, D. C.

It appears that the committee having the matter in charge hope to expend upon the contemplated monument something like $75,000. This seems to be the proper time again to call attention to the fact that this country is the only country in the world where such a proposal could seriously be made, or if made would have the most remote probability of realisation.

With us not only is the proposal obvious and in good taste, but the raising of the money should be an easy matter; in fact, to take less notice of the event, than by building a monument or endowing a hospital, would be for our homeopathists to convict themselves of inconsistency or of ingratitude. However, we who are not likely to be called upon to build this particular monument, might take the opportunity to build another—near at hand—perhaps not quite as large or expensive as the first, but one quite as appropriate and commemorative.

Should it come to pass that the capital of this country has the honor of perpetuating the work of Hahnemann, so far as granite and bronze can so perpetuate it, that honor will be due to the American Medical Association, and it would seem right and proper that that body should claim its own—by balancing the monument to the father of homeopathy, with one to the memory of the *late* N. S. Davis, father of the American Medical Association and foster-mother of homeopathy in America.

I have said that the Davis pile might be smaller than that of Hahnemann, but from this it should not be inferred that the event signalised is of lesser importance or significance; on the contrary, the Davis monument as a record of the greatest piece of malpractice in the history of medicine, and as a warning for all future generations, would have a value of the first order, easily entitling it to a prominent place in the capital of this nation.

HENRY R. HOPKINS,
433 Franklin Street.

BUFFALO, February 22, 1900.

